# The role of glutathione in cognition, cognitive effort, and cognitive endurance in young and older adults

**DOI:** 10.3389/fnagi.2026.1729015

**Published:** 2026-02-27

**Authors:** Geraldine Rodríguez-Nieto, David F. Alvarez-Anacona, Mark Mikkelsen, Hong Li, Stephan P. Swinnen

**Affiliations:** 1 Movement Control and Neuroplasticity Research Group, Biomedical Sciences, KU Leuven, Leuven, Belgium; 2 KU Leuven Brain Institute, KU Leuven, Leuven, Belgium; 3 Universidad Nacional de Colombia, Facultad de Medicina, Bogotá, Colombia; 4 Department of Radiology, Weill Cornell Medicine, New York, NY, United States

**Keywords:** GSH, glutathione, aging, cognition, GABA, glutamate, HERMES, magnetic resonance spectroscopy

## Abstract

Glutathione (GSH) is an abundant antioxidant that protects against endogenous and exogenous toxic agents. The evidence over the relationship between GSH and cognitive integrity during aging is still scarce. In this study we investigated the relationship between GSH and cognitive integrity, cognitive effort and sustained cognitive effort. Second, we explored whether GSH modulation is related to other physiological properties such as blood oxygenation (BOLD response) and to brain excitability (measured by GABA+ and Glx levels). We measured GSH levels through magnetic resonance spectroscopy (HERMES) at baseline and during cognitive task performance in 40 young (18–35 years; 26 female) and 40 older (60–85 years; 21 female) adults in two higher-order processing areas in the brain: the inferior frontal and the inferior parietal cortices (IFC and IPL). GSH in IPL related in opposite directions to distinct memory tasks in young and older adults. GSH levels in both regions showed a modulation as a result of sustained cognitive performance; the direction of this modulation was age- and region-dependent. Furthermore, GSH modulation positively related to cognitive performance in young adults. Finally, GSH showed a relationship with GABA that was region, age and state dependent. These results highlight the heterogeneity of GSH physiology, while its relation with cognition is dependent on age and brain region.

## 1 Introduction

Glutathione (GSH) is an abundant antioxidant and one of its main functions is to protect against toxic agents, such as reactive oxygen species (ROS; Dwivedi et al., 2020). ROS play a role in immunity response and neuroplasticity, but in excess are considered oxidative stressors, posing the risk of causing inflammation and damage to cells (Cheng and Ristow, 2013; Detcheverry et al., 2023; Grimm and Eckert, 2017).

The free radical theory of aging proposes that the damage from oxidative stress plays a key role in age-related diseases (Harman, 1992). Oxidative stress increases with age, can lead to apoptosis, and can affect cognitive integrity (Grimm and Eckert, 2017; Ionescu-Tucker and Cotman, 2021). Therefore, the study of GSH concentration in the brain in the context of aging and its relation with cognitive integrity is mandatory.

Magnetic resonance spectroscopy (MRS) studies show that older individuals have lower GSH levels in the posterior cingulate and occipital cortices (Emir et al., 2011; Hu et al., 2024; Suri et al., 2017), but a null association of GSH with age has also been reported for the posterior cingulate cortex and cerebellum (Gong et al., 2022; van Malderen et al., 2025; Venkateshappa et al., 2012). In contrast, one study reported higher GSH levels in the sensorimotor and frontal cortices of older individuals (Hupfeld et al., 2021). Post-mortem findings showed higher GSH levels in the caudate, cerebellar, and frontal regions (Tong et al., 2016). The current evidence on GSH levels and aging appears very limited and inconsistent, but it is also possible that age-related effects on GSH levels are region dependent.

Some studies in older adults addressed the relationship between GSH levels and general cognitive integrity and found a null association between cognitive integrity and GSH levels in the cingulate, occipital, medial frontal, and sensorimotor cortices (Emir et al., 2011; Hu et al., 2024; Hupfeld et al., 2021). It is possible that the relationship between GSH and cognitive integrity is specific to particular brain regions and/or particular cognitive skills. In older adults with mild cognitive impairment (MCI) GSH levels in both hippocampus and ventromedial frontal cortex were positively correlated with general cognitive integrity but negatively correlated with cognitive flexibility (Mandal et al., 2015). Also in older adults with MCI, higher levels of GSH in ACC were found to be related to lower set shifting and response inhibition, and higher levels of GSH in PCC were related to lower verbal retention (Duffy et al., 2014). Overall, it remains unclear whether executive control skills relate to GSH baseline levels and/or task-related modulation of GSH in regions of the executive network in healthy young and older adults.

Whereas the above-mentioned studies have addressed the relationship of cognitive integrity with baseline levels of GSH, to our knowledge, it has not been explored whether GSH is modulated (showing a decrease or increase) as a consequence of cognitive effort (active engagement in a cognitive process) or sustained cognitive effort (continued engagement during a prolonged period). Functional MRS (fMRS) measures metabolite levels during the performance of cognitive tasks. Different studies have investigated changes in *γ*-aminobutyric acid (GABA) and glutamate (plus glutamine (Glx)) as a consequence of cognitive performance through fMRS (see Li et al., 2024, for a review). Here, we aim to explore whether GSH is modulated as a consequence of cognitive effort, as this involves important physiological activity, such as increased oxygen consumption and changes in neurometabolic activity (e.g., Friston et al., 1994; Li et al., 2024). Further, we aim to explore whether GSH is also modulated as a consequence of sustained cognitive effort, considering that sustained mental activity causes physiological changes that are different from regular cognitive effort (e.g., Barakat et al., 2025; Guillemin et al., 2025), and mental fatigue could possibly induce excessive oxygen consumption and ROS.

Furthermore, it remains elusive whether GSH is upregulated or downregulated as a result of metabolic activity (including blood oxygenation and brain excitability) induced by cognitive effort. We hypothesize that GSH increases during cognitive effort or sustained cognitive effort to reduce the endogenous ROS. In addition, this effect may be enhanced in older adults, as they have a stronger and more diffuse oxygen consumption during cognitive effort (Rodríguez-Nieto et al., 2024). We also hypothesize that a task-induced increase of GSH may be related to an increase of blood oxygenation (as measured by the blood oxygen level-dependent (BOLD) response) and to an increased excitability (shown by higher Glx and/or lower GABA).

First, we aim to investigate the relationship between GSH levels and cognitive features, in particular: (a) age effects on baseline GSH levels in two higher-order brain regions belonging to the executive network (inferior frontal cortex, IFC, and inferior parietal lobe, IPL; Rodríguez-Nieto et al., 2022); (b) the relationship between baseline GSH levels and cognitive integrity, (c) whether GSH levels are modulated by cognitive effort or sustained cognitive effort through fMRS, and (d) whether task-induced modulations in GSH are related to cognitive task performance. Second, we aim to investigate whether GSH levels relate to other local metabolic properties during involvement in cognitive effort, specifically: (e) whether GSH levels are related to blood oxygenation and (g) whether relationships among GSH, GABA and Glx are present at baseline and during task performance.

## 2 Methods

### 2.1 Participants

Forty young and forty older healthy adults participated in this study (young adults (YA): mean ± SD age = 23.38 ± 3.81 years, range = 18–35 years, 26 females; older adults (OA): mean ± SD age = 69.52 ± 5.42 years, range = 60–85 years, 21 females). Participants were recruited through a list of participants of previous studies, through posters and flyers in the university, libraries and social centers and through posts in social media. The gender distribution in both samples was not significantly different from an expected equal distribution, as indicated by a chi-square goodness-of-fit test (Young: X^2^ = 0.783, *p* = 0.37, Old: X^2^ = 0.08, *p* = 0.87; final samples). All participants reported the absence of neurological or psychiatric disorders and were evaluated with the MoCA battery to determine cognitive integrity and to exclude participants with MCI (YA: mean ± SD score = 27.4 ± 3.86, range = 24–30; OA: mean ± SD score = 26.9 ± 2.14, range = 22–30). One older participant scored below the cutoff point (score = 22; cutoff = 24, Islam et al., 2023) and was excluded for that reason. The participants also filled in the Edinburgh Handedness questionnaire. The handedness proportion of participants was representative of that reported for the general population (Willems et al., 2014): 37 young adults and 37 older adults were right-handed; 3 younger adults and 2 older adults were left-handed, and one older adult was ambidextrous. The experiment was approved by the local ethical committee of KU Leuven (protocol number: s61577) and all participants gave written informed consent.

### 2.2 Experimental design

Participants took part in two experimental sessions (scheduled no more than two weeks apart). During the first one, participants filled in self-reports and were tested with the MoCA questionnaire and a battery of Executive Functions (*cognitive integrity* evaluation). During the second session, participants took part in an MRI protocol. In order to investigate whether GSH is modulated by *cognitive effort* and *sustained cognitive effort*, GSH levels were measured in the IFC and in the IPL by MRS at baseline and during task (Rule Switching) performance. These regions were selected according to our meta-analysis results for executive functions paradigms that showed a consistent activation in the IFC and the IPL for the Rule Switching task (Rodríguez-Nieto et al., 2022). Individuals also performed the Rule Switching task during fMRI acquisition prior to the collection of MRS data.

### 2.3 Cognitive integrity

Besides the MoCA test, individuals performed the tests of a Battery of Executive Functions, comprised by: two tests of short-term memory (verbal: Digit Span Forward, spatial: Corsi cubes Forward), two tests of working memory (verbal: Digit Span Backward and spatial: *N*-back test); two tests of inhibition (Go/No-go task and Stroop) and two tests of cognitive flexibility (Wisconsin Card Sorting Test and Number Letter task).

#### 2.3.1 Digit span

Individuals were given a sequence of numbers which they had to repeat. There were two trials for each level (5–7 digits). It was intended that the instructor would mention one cipher per second. The score range was 0–3, where 1, 2 and 3 indicated that the participant correctly repeated 5, 6 or 7 digits. The test stopped when participants failed in the two trials of two consecutive levels. Duration: ~3 min.

#### 2.3.2 Digit backward

Participants were given a sequence of numbers as in the Digit Span, but they had to mention the sequence in reverse order (e.g., sequence: 1–3–5, individual’s correct answer: 5–3–1). The instructor mentioned one cipher per second. The score was the number of ciphers in the most difficult level, where participants had two trials to complete correctly. The test stopped when participants failed in the two trials of two consecutive levels. Duration: ~3.5 min.

#### 2.3.3 Corsi cubes

Seven white squares were displayed on a gray background on the computer screen. Participants would see these squares lit up in blue one by one in a sequence of 1 to 7 items (seven trials respectively). After the sequence finished (indicated by an asterisk on the screen), participants had to click on the cubes in the same order as they lit up. The score was the most difficult trial, where participants repeated the sequence in the correct order. Duration: ~5 min.

#### 2.3.4 N-back

Twelve squares were presented on the screen. These squares would light up one by one in a randomized order. Each time a square lit up, participants had to indicate whether that square was the same one that was lit two trials before. There were six blocks with 24 trials for each block. The score was the number of correct trials. Duration: ~5.5 min.

#### 2.3.5 Go/No-go

Participants would see either a “C” or an “M” at the center of the screen. They were instructed to press the spacebar when they saw the “C” (which represented 75% of the trials) and not to respond to the “M” (25% of the trials). Each stimulus lasted 650 ms. There were four blocks with 40 trials. The score was the number of False Alarms; this is the number of trials where individuals pressed the spacebar in the No-go trials (trials with an “M”). Duration: ~6 min.

#### 2.3.6 Number Stroop task

Participants would see numbers with 1–4 ciphers (where each cipher was the same number, which could be 1, 2, 3, or 4, e.g., 22, 3, 444). Participants had to indicate how many ciphers they saw, regardless of the number shown (e.g., for 444, they had to indicate ‘3’). Half of the trials were congruent (24/48), where the numbers shown matched the number of ciphers (e.g., 333) and half of them were incongruent (e.g., 222). The score was the number of hits (successful trials) in the incongruent condition subtracted from the number of hits in the congruent condition, representing the inhibition cost. Duration: ~3.5 min.

#### 2.3.7 Wisconsin Card Sorting Test (WCST)

Participants were instructed to sort cards according to three possible categories (color, shape or number). The rule was not explicit, but participants received feedback after each classified card (correct or incorrect). The classification rule switched after seven trials (without notifying the participant). There were two blocks, each one including 192 trials (64 for each condition: shape, color or number). Duration: ~5.5 min.

#### 2.3.8 Number letter task (NLT)

In each trial, a pair of one letter and one number was shown in one out of four quadrants of a square. If the pair appeared in one of the two top quadrants, participants had to categorize the stimulus according to the number from the pair, indicating whether it was odd or even through button press (‘z’ or ‘/’ keys). If the pair appeared in one of the two bottom quadrants, participants had to indicate whether the letter from the pair was a consonant or a vowel. To reduce the load of working memory—as this task is meant to measure cognitive flexibility—participants first performed the number and letter tasks separately (34 trials for each one). Afterwards, they performed the NLT (two blocks with 68 trials each). Switch trials involved switching the rule in reference to the previous trial, whereas repeat trials involved maintaining the same rule as in the previous trial. The score was the number of hits in switch trials subtracted from the number of hits in repeat trials, representing the switching cost. Duration: ~12 min.

### 2.4 MRI task protocol

**Figure 1:**
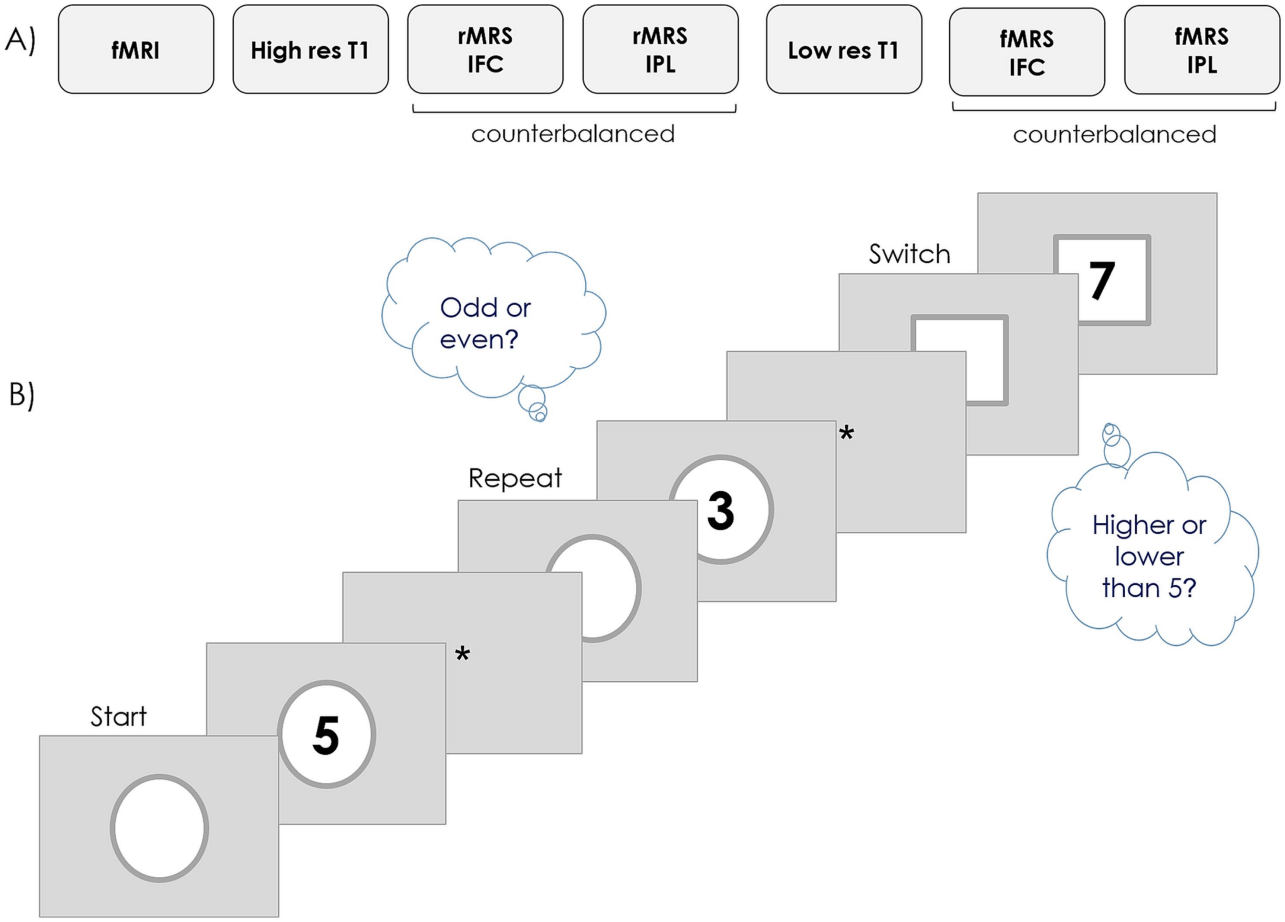
Schema of the experimental protocol. **(A)** MRI protocol: rMRS, resting state MRS; fMRS, task-related MRS. **(B)** Rule switching task.

Prior to the MRI session, participants performed 16 practice trials of the Rule Switching task used to assess cognitive flexibility. Once positioned in the MRI scanner, they performed the Rule Switching task during the fMRI acquisition session (~6 min). Afterwards, the MRS protocol consisted of: (a) a high-resolution anatomical sequence (~6 min; placed after the fMRI to avoid the heat induced by the fMRI sequence to affect the quality of the MRS acquisition), (b) two MRS scans at baseline (resting-state MRS; ~12 min each) with the voxels centered at the IFC and IPL, (c) a short *T*_1_-weighted scan to minimize the effect of intermediate head movement on voxel location by using it for the co-registration of images collected during the MR session (~ 3 min), and (d) two MRS scans with the voxels centered at the IFC and IPL while participants performed the task (task-related functional MRS; ~12 min each) (Figure 1A). The order of the two MRS scans in (b) and (d) was counterbalanced across participants.

### 2.5 Cognitive performance and sustained cognitive effort (task performance during MRI)

In the Rule switching flexibility task (adapted from Sekutowicz et al., 2016; launched in PsychoPy, Peirce, 2007), participants were asked to respond using a button box whether the number shown on the screen was lower or higher than five (left or right button) when the number was preceded (cue) and simultaneously surrounded by a circle and to indicate whether it was odd or even (left or right button) if the number was preceded (cue) and simultaneously surrounded by a square (Figure 1B). In half of the trials, the rule was the same as in the previous trial (Repeat trial), and in the other half, the rule switched (Switch trial) based on a pseudo-randomized sequence. During fMRI, the task consisted of 94 trials (cue alone = 300 ms; cue + stimulus = 1700 ms; ITI = 3,000 or 4,000 ms). During MRS, the task consisted of 132 trials in each of two blocks (cue alone = 400 ms; cue + stimulus = 2,200 ms; ITI = 2000, 3,000, or 4,000 ms).

For this task, we calculated the cognitive flexibility index by subtracting the number of hits in Repeat trials from the number of hits in the Switch trials. This estimation was done with the data from the fMRI block to avoid the effects of learning and fatigue on cognitive flexibility. A higher score indicated a better cognitive flexibility performance. One old participant was excluded due to a very low flexibility score (< 3 SD).

### 2.6 Imaging data acquisition

MRI scans were acquired using a Philips Achieva 3 T scanner with a 32-channel head coil. For structural imaging, a high-resolution three-dimensional *T*_1_-weighted image was collected (MPRAGE, TR/TE/TI = 9.6/4.6/900 ms; 0.98 × 0.98 × 1.2 mm^3^ voxel size; FOV = 192 × 250 × 250 mm^3^; 160 coronal slices, scan time ∼7 min). Before the task-related MRS, a short *T*_1_-weighted anatomical image was acquired to verify correct alignment for the MRS voxels.

#### 2.6.1 fMRI

To acquire fMRI scans, an ascending gradient EPI pulse sequence for *T*_2_*-weighted images (TR/TE = 1000/33 ms; flip angle = 70°; 42 transverse slices; interslice gap = 0.5 mm; voxel size = 2.15 × 2.14 × 3 mm^3^; FOV = 240 × 240 × 146.5 mm^3^) was used.

#### 2.6.2 fMRI data analysis

fMRI data were analyzed using the FMRIB Software Library (FSL) v.6.05. First, the Brain Extraction Tool (BET) was used on the *T*_1_-weighted anatomical images to extract the brain from the dura and skull. Next, the fMRI data were preprocessed: data were high-pass filtered with a cut-off time constant of 90 s, motion-corrected with MCFLIRT, and spatially smoothed at 8-mm full-width half-maximum (FWHM). Functional images were co-registered to their corresponding *T*_1_-weighted anatomical image using a linear transformation (12 DOF) and visually inspected. The resulting image was normalized (linear registration, 12 DOF) to the MNI152 template. fMRI data from six YA participants were excluded due to technical difficulties resulting in unusable data (four) and excessive head movement (two; cutoffs: 1.5 mm for absolute and 0.65 for relative motion).

For each individual, we applied a fixed-effects GLM in which Repeat trials, Switch trials and inter-trial intervals were included as regressors along with their first temporal derivative. The statistical maps were rendered using a *z*-threshold of >2.3 and a (corrected) cluster significance threshold of *p* = 0.05. The motion parameters were included as regressors in the first-level GLMs.

A random-effects model was used to extract each group’s mean BOLD activity in the Switch > ITI contrast. Next, group comparisons OA > YA and YA > OA were performed for this contrast (maps were cluster-corrected at a threshold of *p* = 0.05 and rendered using a *z*-value >2.7).

The beta values from the Switch > ITI contrast were extracted for each participant from a mask delineated from the sum of the individual IFC and IPL voxels, to examine the relationship between BOLD and GSH levels.

#### 2.6.3 MRS

For MRS, a Hadamard Encoding and Reconstruction of Mega-Edited Spectroscopy (HERMES; Saleh et al., 2016) sequence was used: TR/TE = 2000/80 ms; 320 water-suppressed transients, 5 kHz spectral width; 4,096 datapoints, 16 water-unsuppressed transients. The HERMES sequence makes possible the measurement of GABA, Glx and GSH from the same MRS block, preserving the quality and signal to noise ratio of the MEGA-PRESS sequence (Saleh et al., 2016). In addition, this sequence has shown to have good reproducibility for the measurement of GABA, GSH and Glx (DeMayo et al., 2025). This study applied real-time frequency correction based on interleaved water referencing (Edden et al., 2016). Water suppression was achieved using the ‘VAPOR’ sequence (Tkáč et al., 1999).

Four MRS blocks (two conditions × two voxels) were conducted to target GSH levels in the left IFC (4 × 2.5 × 2.5 cm^3^ voxel) and in the left IPL (3 × 3 × 3 cm^3^ voxel).

As the acquisition of a HERMES sequence after an fMRI or DWI sequence may be comprised by a frequency drift derived from the heating of the scanner, we aimed to prevent that problem by the use of a short fMRI sequence (7 min) and by acquiring the T1 sequence between the fMRI and the MRS. The T1 sequence acquisition together with the voxel positioning for the MRS had a joint duration of ~9 min. A visual inspection of the creatine frequency plots, indicated that this procedure was successful in avoiding frequency drifting.

The positioning of these voxels was selected to optimize overlapping with the neural maps from our previous meta-analysis on executive task paradigms (including Rule Switching; Rodríguez-Nieto et al., 2022). The procedure to locate the voxel for the IFC was: (a) center the voxel at the first axial slice above the ventricles at the height of one third of the sagittal midline and at the width center of the left hemisphere, (b) from the sagittal view locate the center length of the voxel parallel to the inferior frontal sulcus and locate the ¾ length over the inferior frontal junction, and (c) from the coronal view, locate the top of the voxel parallel to the cortical surface. When needed, this procedure was adapted to avoid covering the ventricles or the subarachnoid space. To locate the IPL voxel we: (a) located the voxel four slices above the last axial view of the ventricles and the first view after the ventricles in the coronal plane, (b) placed one side parallel to the occipito-parietal sulcus, (c) moved the voxel toward the axial midline of the left hemisphere and (d) in the sagittal plane, located it parallel to the inferior parietal sulcus and inspected that this was mostly covered.

### 2.7 MRS data analysis

Data were preprocessed and quantified using Gannet v3.3.0 (Edden et al., 2014). Preprocessing steps included line-broadening (3 Hz), eddy-current correction, robust spectral registration (Mikkelsen et al., 2020), signal averaging, residual water filtering and reconstruction of subspectra to generate a GSH-edited spectrum. All preprocessed data were visually inspected to check for spectral artifacts and to assess the spectral data quality. Nine GSH measurements (three OA from the IFC baseline, two OA from the IPL baseline, three OA from the IFC task condition, and one YA for the IFC task) were excluded for a fitting error (defined as the standard deviation of the model residuals divided by the amplitude of the fitted GSH signal) above 3 SD. Two datapoints from GABA+ (GABA plus macromolecules) in the IFC task condition were excluded for the same reason (presenting a fitting error above 3 SD).

**Figure 2:**
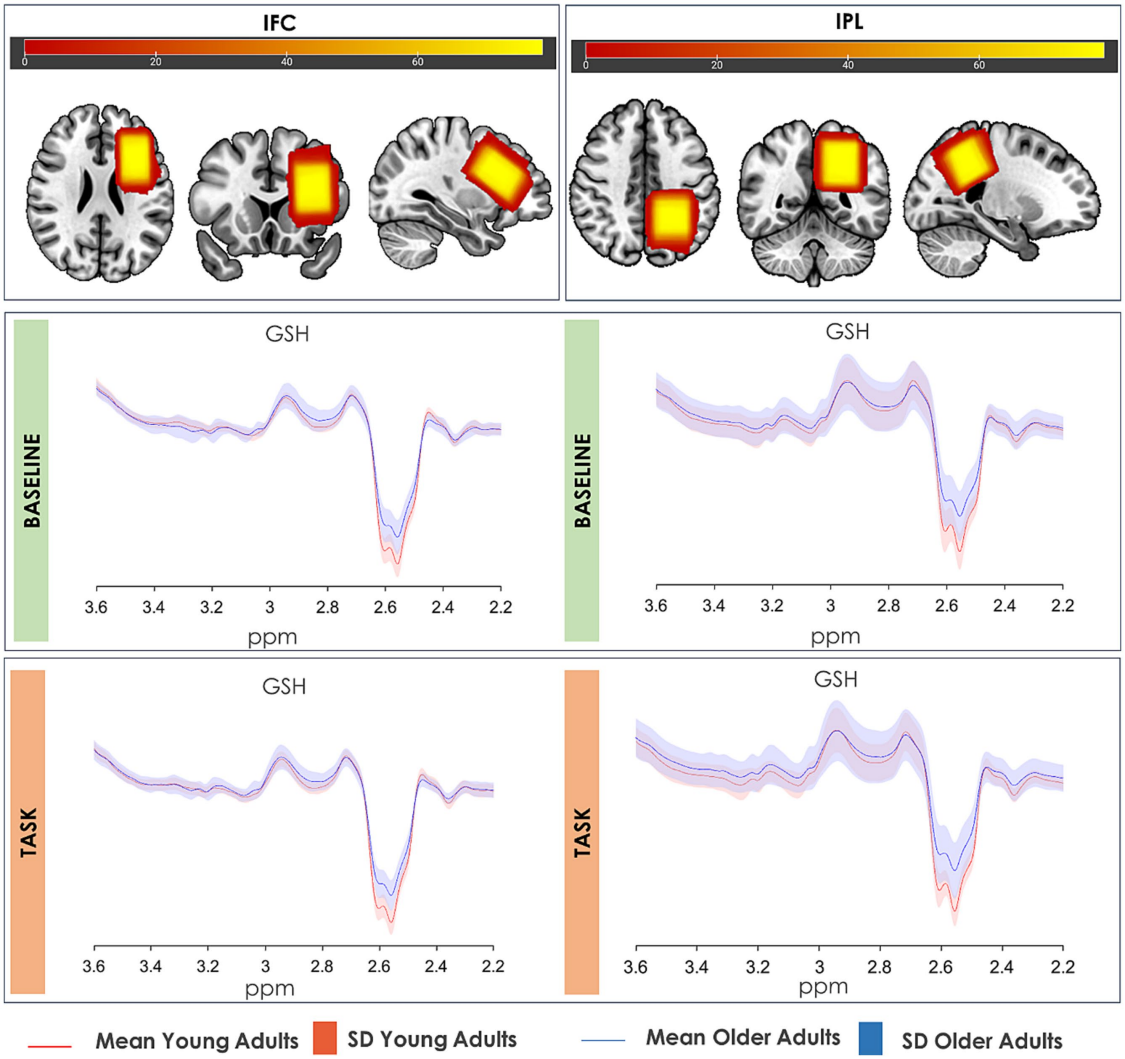
Group-average GSH-edited HERMES spectrum. GSH average spectrum of young and older individuals in the IFC (left) and the IPL (right) during resting-state MRS (top) and task-related MRS (bottom). The overlap of the full sample voxels for each region is shown in the top panel. Shaded colors represent the standard deviation of the spectrum.

The voxel masks for each analysis were co-registered to their respective anatomical image to determine gray matter, white matter and cerebrospinal fluid fractions using SPM12 (Friston et al., 1994). The metabolite levels were quantified in institutional units (i.u.) using tissue water as an internal concentration reference. GABA+ and Glx were tissue-corrected according to the alpha-correction method of Harris et al. (2015), where the WM: GM concentration ratio was assumed to be 1:2. For GSH, the ratio was assumed to be 1:1, as there are currently no studies demonstrating a bulk tissue dependence for MRS-measured GSH. Figure 2 shows the overlap of the voxels from all participants positioned for IFC and IPL, as well as the group-average spectrum for each condition (baseline and condition) and by age group, highlighting consistent voxel placement and excellent spectral quality. A group-average spectrum of GABA+ and Glx from this sample has been displayed in our previous report (Rodríguez-Nieto et al., 2024).

### 2.8 Statistical analyses

#### 2.8.1 Effect of age and cognitive performance on GSH concentrations

A mixed GLM model (repeated measures) was conducted to address differences in GSH according to Age group (YA vs. OA; inter-subject variable) and Condition (Baseline vs. task; Intrasubject variable) in IFC and IPL, with the order (measured during first or second block) as a covariate.

#### 2.8.2 Relationship between GSH levels and cognitive integrity

We performed normality tests to determine whether to use Spearman or Pearson correlations. We conducted correlations among GSH baseline levels in IFC and IPL, and the total MoCA score, as well as each score from the Battery of Executive Functions tasks. Afterwards, a multiple comparison correction (Benjamini–Hochberg method) was applied to the correlations.

#### 2.8.3 Effect of sustained cognitive effort on GSH levels (GLM with age and order)

A 2 × 2 GLM was used to assess the effect of sustained cognitive effort by using Age and Order as inter-subject variables. For Order, we considered the participants where the specific VOI (IFC or IPL) had been measured first or second from the counterbalanced design. In this manner we compared participants that performed the task for 12 min with those who performed the task for 24 min (according to the region in the counterbalanced protocol). This model was conducted separately for each VOI.

#### 2.8.4 Relationship between task-induced GSH modulation and cognitive performance

We estimated a GSH modulation index (GSH baseline levels subtracted from GSH levels measured during the task), and we entered the GSH modulation index from IFC and IPL as predictors in a regression model with the cognitive flexibility index as a dependent variable. As we knew *a priori* about age differences in the distinct variables, we executed a regression model for each group.

#### 2.8.5 Relationship between task-induced GSH modulation and blood oxygenation (BOLD response)

To assess whether GSH modulation (GSH baseline levels subtracted from GSH levels measured during the task) demonstrated a relationship with the blood oxygenation, we conducted correlations among these variables and the beta parameter extracted from the fMRI BOLD signal (Contrast: Switch > ITI).

#### 2.8.6 Relationship between GSH and brain excitability (GABA+ and Glx)

A graph theory approach was used to examine the relationship between GSH, GABA+ and Glx in three conditions: (a) baseline, (b) task and (c) modulation (metabolic change from baseline to task). This approach was previously described in Rodríguez-Nieto et al. (2023). In sum, a graph is created from the correlation matrix (after multiple-comparison correction) of different metabolites. Each metabolite constitutes one node in the graph, and the edges represent the presence of a relationship among them. As the information from other metabolites was available too (choline and creatine), this information was included for complementary purposes and allows comparability with previous observations (Rodríguez-Nieto et al., 2023).

## 3 Results

### 3.1 Effect of age and cognitive performance on GSH concentrations

The mixed GLM model revealed an Age effect on the GSH levels from IFC with GSH being higher in older individuals regardless of the condition [*F*_(1,74)_ = 13.01, *p* < 0.001; YA: mean = 1.28, SD = 0.33; OA: mean = 1.73, SD = 0.86]. The same model applied for the IPL, revealed no age differences [Group: *F*_(1,76)_ = 0.75, *p* = 0.39; YA: mean = 1.97, SD = 1.03; OA: mean = 2.14, SD = 0.87].

**Figure 3:**
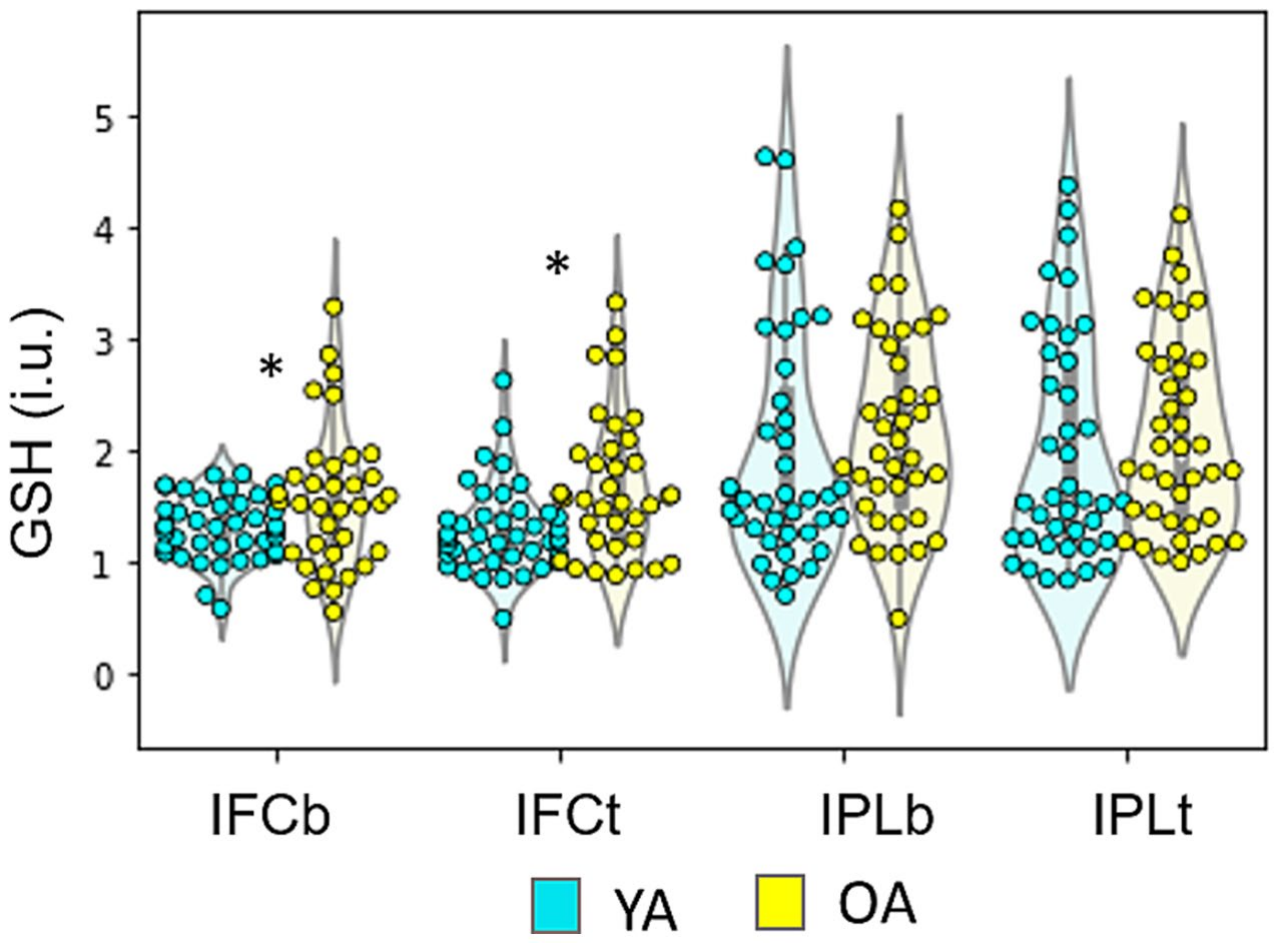
GSH levels in both age groups across baseline (b) vs task (t) conditions. IFC – inferior frontal cortex, IPL – inferior parietal lobe. Older adults (OA) had significantly higher levels of GSH in the IFC (regardless of the condition) compared to younger adults (YA) [Age X Condition ANOVA; *F*_(1,74)_ = 13.01, *p* < 0.001]. No differences between the two age groups were observed in the IPL [*F*_(1,76)_ = 0.75, *p* = 0.39] and no effect of condition (baseline vs. task) was found, nor an interaction in either region (IFC: Condition: *F*_(1,74)_ = 0.02, *p* = 0.89; Interaction: *F*_(1,74)_ = 0.07 *p* = 0.78; IPL: Condition: *F*_(1,76)_ = 0.16, *p* = 0.69; Interaction: *F*_(1,76)_ = 0.07, *p* = 0.78).

There were no significant differences on GSH levels as an effect of cognitive performance (Rule Switching task), nor its interaction with age in IFC [Condition: *F*_(1,74)_ = 0.02, *p* = 0.89; Interaction: *F*_(1,74)_ = 0.07 *p* = 0.78] or in IPL [Condition: *F*_(1,76)_ = 0.16, *p* = 0.69; Interaction: *F*_(1,76)_ = 0.07, *p* = 0.78; Figure 3].

### 3.2 Relationship between GSH levels and cognitive integrity

**Figure 4:**
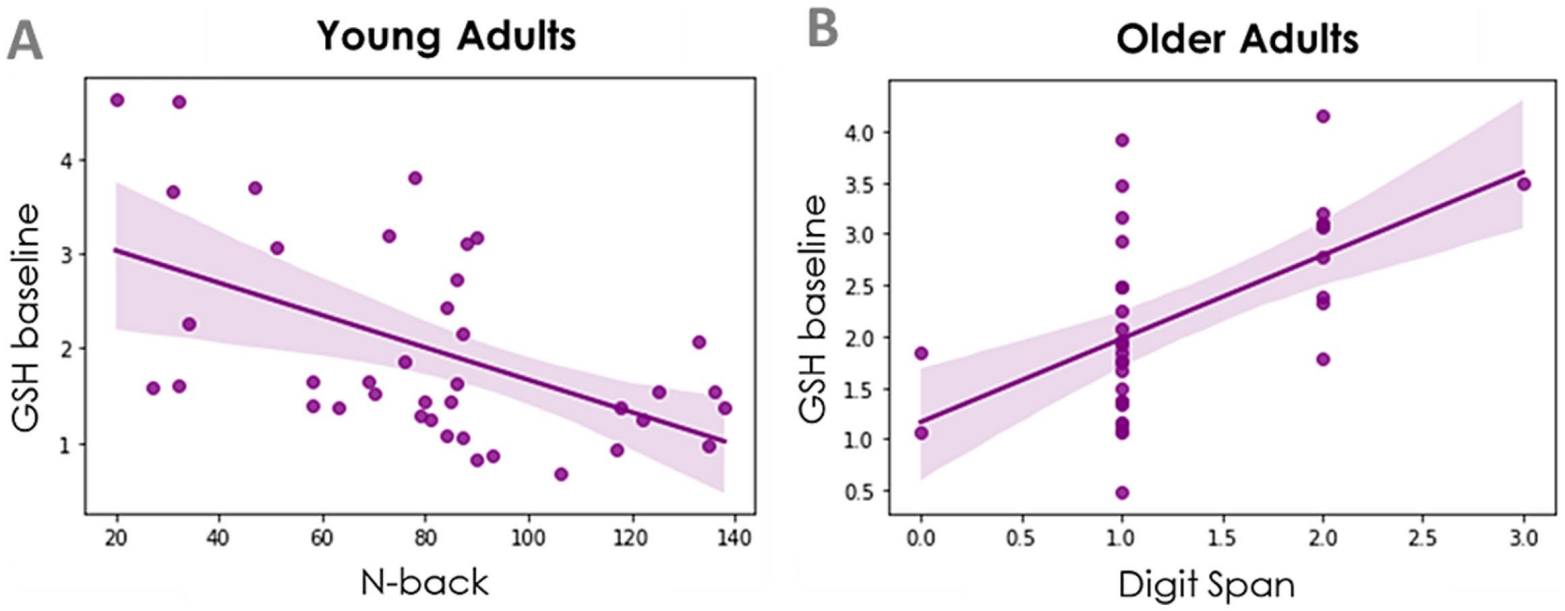
Relationship between baseline GSH in the IPL and cognitive performance. **(A)** In young adults, a better performance in the N-back task was related to lower baseline levels of GSH in the IPL (Pearson coefficient = −0.53 *p* < 0.001, *n* = 39). **(B)** In older adults, higher baseline levels of GSH were related to a better performance in the digit span task (Spearman coefficient = 0.56, *p* < 0.001, *n* = 35).

The correlations between baseline GSH levels and the MoCA and the tests from the Battery of Executive Function are presented in Table 1. Only two correlations survived multiple comparisons correction. The first one showed that lower baseline levels of GSH in the IPL were related to a better performance in the *N*-back task in young adults (Figure 4A). The second correlation showed that higher baseline levels of GSH in the IPL were related to a better performance in the Digit Span task in older adults (Figure 4B).

**Table 1:** Correlations between baseline GSH and cognitive performance tests for both brain regions and age groups.

Group	GSH (baseline)	MoCA total^s^	Digit span^s^	Corsi forward^s^	Digit backward^s^	Nback^p^	Go/No- Go^p^	Stroop^s^	Corsi inverse^s^	WCST^s^	NLT^s^
Young	IFC	0.24 (0.12)	0.06 (0.71)	0.29 (0.07)	−0.08 (0.63)	−0.03 (0.86)	0.16 (0.32)	0.34 (0.03)	0.11 (0.51)	0.32 (0.04)	0.02 (0.89)
	IPL	−0.01 (0.92)	0.22 (0.18)	0.13 (0.42)	0.08 (0.61)	−0.53 * (<0.001)	0.16 (0.34)	0.11 (0.52)	0.02 (0.91)	0.08 (0.61)	−0.01 (0.92)
Old	IFC	−0.01 (0.93)	−0.01 (0.99)	−0.06 (0.73)	−0.16 (0.35)	−0.11 (0.51)	0.09 (0.59)	0.01 (0.99)	−0.07 (0.69)	−0.01 (0.91)	−0.13 (0.43)
	IPL	0.21 (0.22)	0.56 * (<0.001)	0.01 (0.97)	0.21 (0.23)	−0.06 (0.72)	−0.03 (0.86)	0.09 (0.61)	−0.16 (0.34)	0.29 (0.08)	0.24 (0.16)

^*^Indicates the values that survived multiple comparison correction with the method of Benjamini–Hochberg. ^s^ and ^p^ point to Spearman and Pearson correlations, respectively.

### 3.3 Effect of sustained cognitive effort on GSH levels

**Figure 5:**
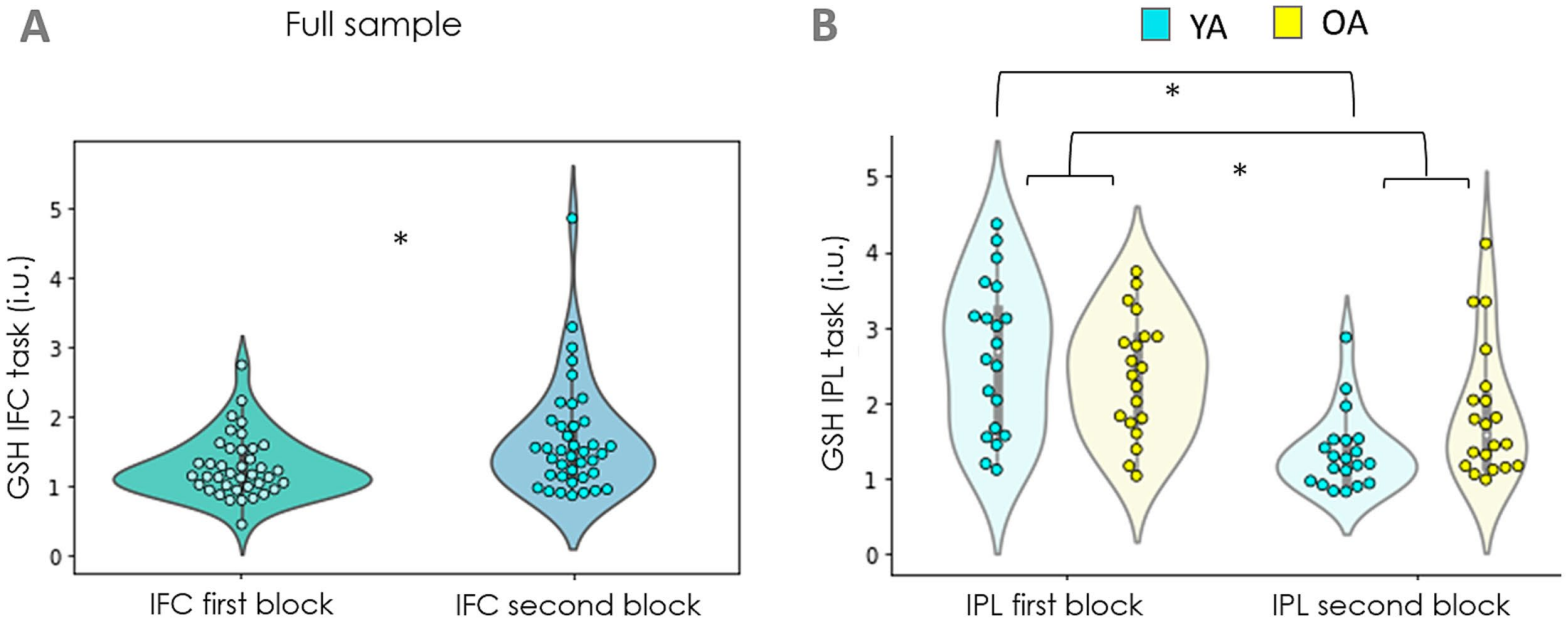
Effect of sustained cognitive effort on GSH levels. **(A)** GSH levels in the IFC were higher when they were measured during the second task block (i.e., during sustained cognitive effort) in the full sample [*F*_(1,74)_ = 4.59, *p* = 0.03]. **(B)** GSH levels in the IPL were lower during the second task block in the full sample [*F*_(1,76)_ = 23.99, *p* < 0.001] and this difference was sharper in the young adults sample [*F*_(1,76)_ = 4.53, *p* < 0.04].

The GLM revealed order effects in both regions [IFC: R^2^ = 0.18, *F*_(1,77)_ = 4.59, *p* = 0.035; IPL: R^2^ = 0.27, *F*_(1,79)_ = 23.96, *p* < 0.001]. This implied that the GSH levels were higher during the second task block in IFC but lower when measured in the second task block in IPL in the full sample (Figures 5A,B; Table 2). Furthermore, the order effect showed a significant interaction with age in the IPL but not in the IFC [IFC: *F*_(1,77)_ = 0.13, *p* = 0.71; IPL: *F*_(1,79)_ = 4.52, *p* < 0.04]. In particular, the sustained effort had a stronger influence (accompanied by a steeper decrease) on GSH levels in IPL in younger individuals (Figure 5B; Table 2).

**Table 2:** Descriptive statistics of task-related GSH levels during cognitive performance according to order of presentation.

GSH IFC task	IFC first block Mean (SD) Range	IFC second block Mean (SD) Range
Young	1.16 (0.26) 0.48–1.69	1.41 (0.47) 0.86–2.62
Old	1.56 (0.55) 0.87–2.85	1.92 (0.97) 0.91–4.91

GSH IPL task	IPL first block Mean (SD) Range	IPL second block Mean (SD) Range
Young	2.62 (1.004) 1.12–4.37	1.34 (0.51) 0.83–2.87
Old	2.37 (0.79) 1.04–3.74	1.87 (0.87) 0.99–4.11

To assess whether this effect would simply be due to a natural fluctuation in time, we performed similar analyses for the baseline condition, finding no significant order effects [IFC: R^2^ = 0.11, *F*_(1,79)_ = 0.75, *p* = 0.39; IPL: R^2^ = 0.04, *F*_(1,78)_ = 1.21, *p* = 0.28] nor an interaction with Age [IFC: *F*_(1,79)_ = 0.01, *p* = 0.96; IPL: R^2^ = 0.05, *F*_(1,78)_ = 0.92, *p* = 0.34; Supplementary Table S1; Supplementary Figure S1]. In addition, for complementary purposes, and to explore how this pattern compares to other metabolites, we performed similar analyses for GABA+ and Glx. In sum, GABA+ levels in the IPL of the full sample were lower in the second block of baseline but higher during the second block of the task, whereas Glx levels in the IPL of young individuals were lower during the second block of baseline (Supplementary Tables S2–S4; Supplementary Figures S2, S3).

### 3.4 Relationship between task-induced GSH modulation and online cognitive performance

**Figure 6:**
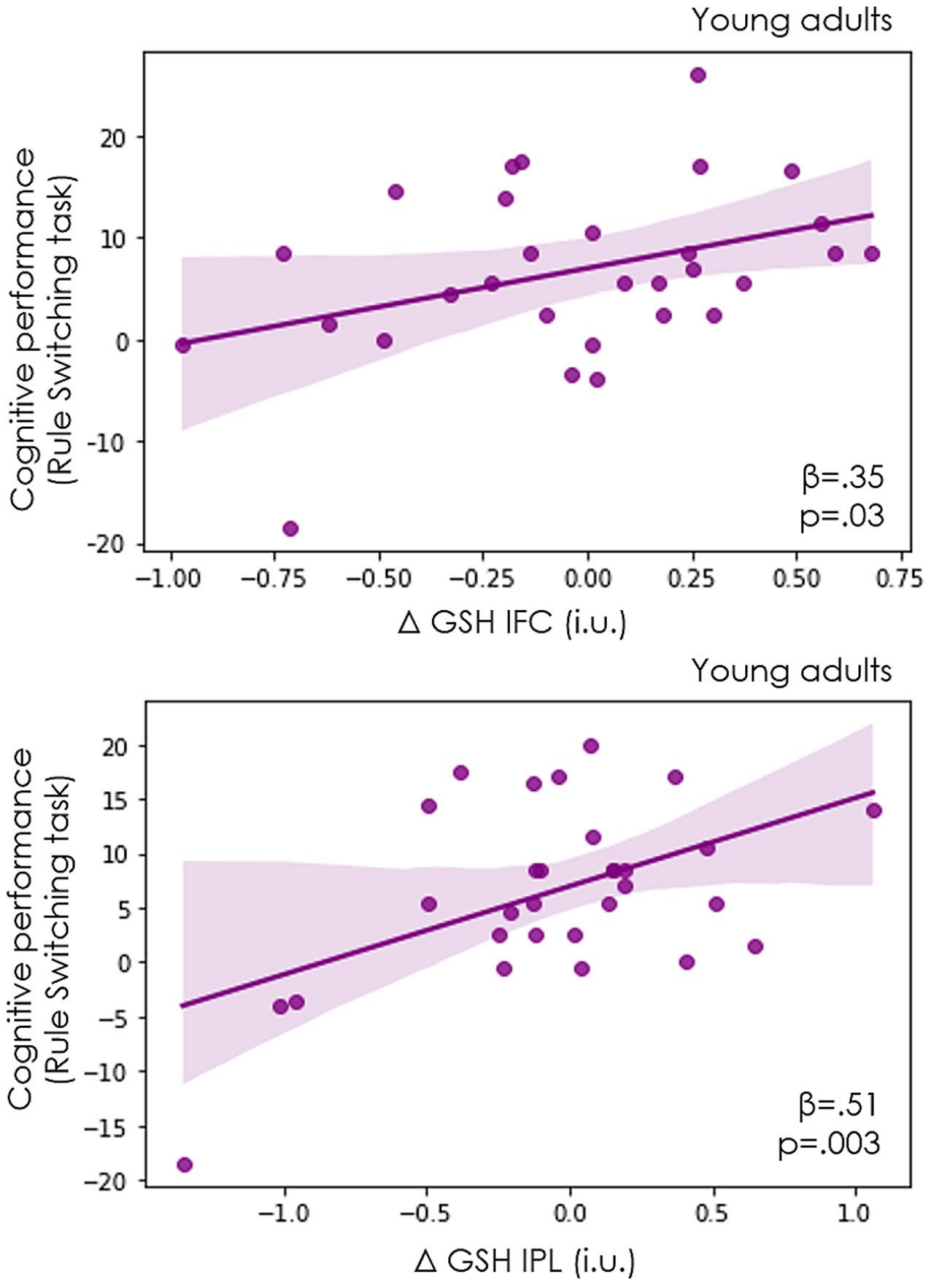
Correlations between GSH modulation and cognitive performance in young adults. In young adults an increase in GSH levels in IFC (top) and IPL (bottom) during task performance (as compared to baseline levels) predicted a better performance in the rule-switching task (*n* = 39).

In young adults, the regression models showed that the modulation of GSH in both regions predicted cognitive performance, showing that an increase in GSH was accompanied by a better performance in the Rule Switching task (R^2^ = 0.39, *F*_(31,2)_ = 8.21, *p* = 0.002; ΔGSH IFC *β* = 0.35, *t* = 2.31, *p* = 0.03; ΔGSH IPL *β* = 0.51, *t* = 3.32, *p* = 0.003; Figure 6). In contrast, none of these variables predicted cognitive performance in the older adults group (R^2^ = 0.065, *F*_(35,2)_ = 0.83, *p* = 0.44; ΔGSH IFC *β* = −0.18, *t* = −1.07, *p* = 0.28; ΔGSH IPL *β* = 0.11 *t* = 0.58, *p* = 0.56).

These relationships remained significant after controlling for local levels of GABA+ modulation (see section on the relationship of GSH with GABA+ and Glx; IFC r = 0.35, *p* = 0.005; IPL r = 0.52, *p* = 0.004).

### 3.5 Relationships between task-induced GSH modulation and blood oxygenation (BOLD response)

**Figure 7:**
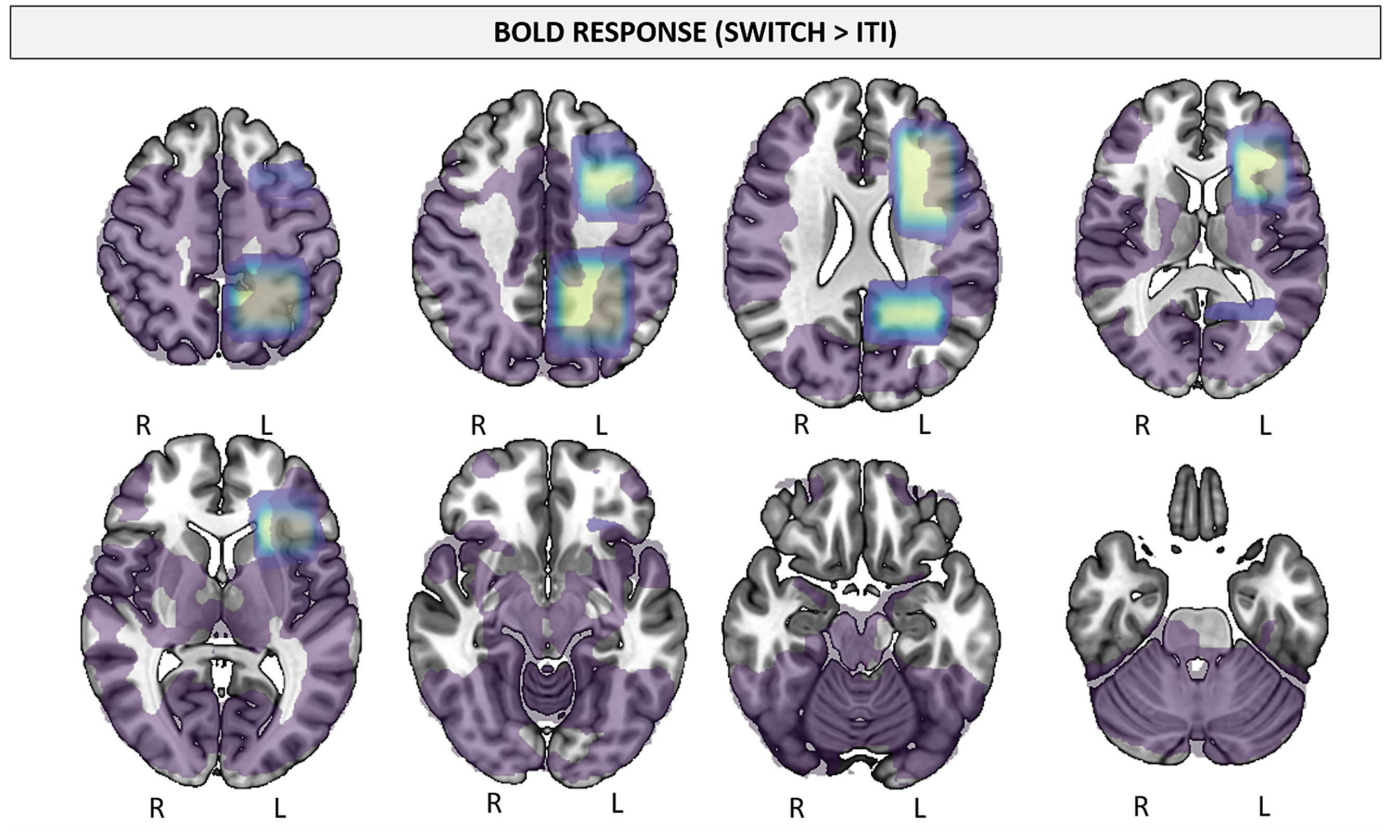
Bold response in the full sample (Switch > ITI; *z* = 2.7; cluster significance threshold *p* < 0.05).

The relationship between task-induced GSH modulation and the BOLD response (within the respective voxel) during cognitive effort was not significant in either group (Table 3). The whole-brain BOLD response is displayed in Figure 7 for the full sample (no age differences were observed in the Switch>ITI contrast).

**Table 3:** Relationship between task-induced GSH modulation and BOLD response during cognitive performance.

Group	BOLD IFC	BOLD IPL
Young (local GSH)	0.14^s^ (0.43)	0.13^s^ (0.46)
Old (local GSH)	−0.03^s^ (0.83)	−0.02^p^ (0.89)

^s^Indicates Spearman coefficient; ^p^indicates Pearson coefficient. *p* values are shown in brackets.

### 3.6 Relationship between GSH and brain excitability (GABA+ and Glx)

**Figure 8:**
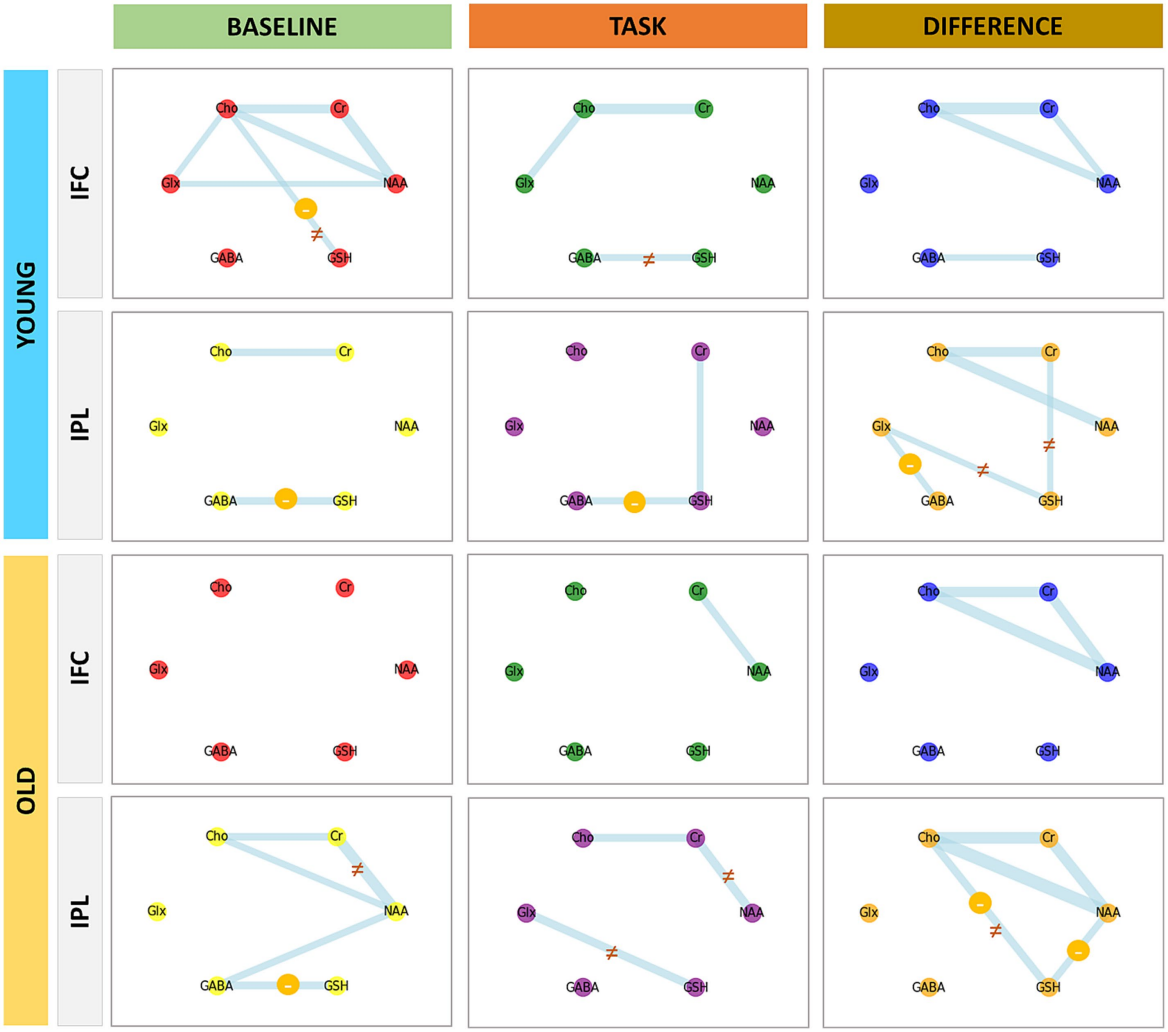
Relationship between GSH and other metabolites across age groups, VOIs, and conditions. Graph theory networks showing the relationship among metabolites during rest and during the task (cognitive performance). The difference refers to the modulation of such metabolites (task levels minus rest levels). The (−) symbol in yellow circles indicates a negative relationship. The (≠) symbol indicates the correlations that were significantly different between young and older adults.

The graph theory analyses revealed that GSH in IPL correlated negatively with GABA+ during rest in both age groups. GSH showed a positive correlation with Glx in the IPL only in older adults during the task performance. In addition, the GABA+ modulation (change from baseline to task performance) correlated with the GSH modulation in IFC in young adults, implying a co-variation of both metabolites as a product of cognitive performance (Figure 8). The correlation matrices for these analyses and the comparison of significant correlations between young and older adults can be found in Supplementary Tables S7–S11.

## 4 Discussion

In this study, we aimed to investigate the association between age and GSH levels in two critical higher-order processing brain regions involved in executive functions. We also determined the relationship between GSH levels and cognitive integrity, cognitive performance and sustained cognitive effort. Finally, we investigated whether GSH levels were related to blood oxygenation and to the levels of GABA+ and Glx during cognitive effort. We found that: (a) GSH was higher in older adults only in the IFC, (b) there was a relationship between GSH baseline levels and short-term memory and working memory in young and older individuals respectively, yet with opposite directions, (c) at the group level there were no significant GSH changes as a result of cognitive task performance in neither region nor group, (d) there was an effect of sustained cognitive effort on GSH levels, which was dependent on the brain region and (e) the task-induced GSH modulation in both regions predicted cognitive performance only in young adults. In relation to metabolic properties, we observed: (f) a lack of relationship between task-induced GSH modulation and blood oxygenation, and (g) a negative correlation between GSH and GABA+ in IPL. Next, these findings are discussed in depth.

### 4.1 Relationship between GSH and age

GSH levels were higher in older adults in the IFC, but no age differences were observed in the IPL. This observation is consistent with the suggestion that the age-GSH relationship may be brain-region dependent (Detcheverry et al., 2023). This finding is also consistent with one MRS and one post-mortem study that showed higher GSH levels in frontal cortices in older adults (Hupfeld et al., 2021; Tong et al., 2016). As protein oxidative damage and decline in oxidative stress homeostasis occur in the frontal lobe during aging (Cabré et al., 2017) and the frontal lobes exhibit the greatest age effects in the brain (e.g., gray matter reduction and volume loss; Raz et al., 1997; Salat et al., 2004; Jernigan et al., 2001), it is possible that higher GSH frontal levels emerge as an adaptive response.

### 4.2 Relationship between baseline GSH levels and cognitive integrity

We did not observe a relationship between GSH levels in either region and general cognitive integrity as measured by the MoCA. However, higher GSH levels in the IPL were related to worse working memory (*n*-back) in young individuals but to higher short-term memory (Digit Span) in older individuals. The latter finding mildly resembles the study from Hu et al. (2024), who reported a positive correlation between GSH levels in the posterior cingulate cortex (but not from the anterior cingulate cortex) and performance in a visuospatial memory task in a sample of individuals 20–70 years old; although the difference in age range of the sample and the brain region studied makes difficult a direct comparison. The IPL processes numerical and visuospatial information (Liu et al., 2021), skills being tapped by the N-back and Digit Span tasks. The GSH-cognition relationship with opposite patterns in both age groups suggests that GSH levels (in IPL) may be indicative of different underlying processes in young and older adults. Whereas higher levels of GSH in the IPL in younger adults may be indicative of excessive oxidative stress (impairing cognitive processing), in older adults higher GSH levels in the IPL may be indicative of a healthy adaptive antioxidant response. The reason why the relationship between GSH and cognitive performance was specific to the IPL remains to be elucidated.

### 4.3 Effect of cognitive effort and sustained cognitive effort on GSH levels

The cognitive effort (or task performance) per se did not have any significant effect on GSH levels in either region or either age group. Nonetheless, sustained cognitive effort affected GSH levels, and, once more, this effect was region dependent. In the full sample, GSH in the IFC was higher during sustained cognitive effort. However, in the IPL, GSH levels were lower during the same condition (sustained cognitive effort), with this effect being stronger in young individuals. Two key yet distinct phenomena may play a role during sustained cognitive effort: automatization and/or fatigue processes. Whereas automatization implies that a particular region requires fewer neurometabolic (but more finely tuned) resources, fatigue implies a continuous effort accompanied by a sustained use of resources or even resource depletion. Such continuous cognitive effort, accompanied by a sustained metabolic activity, may eventually induce endogenous oxidative stress (through a sustained generation of ROS during energy production; see Grimm and Eckert, 2017) and evoke a consequent GSH modulation. We had previously hypothesized that the IFC may be more active in the initial phases of rule switching, whereas the IPL may be continuously engaged (Rodríguez-Nieto et al., 2024). Under this reasoning, lower levels of GSH in the IPL during the second block could be interpreted as a depletion of available GSH, whereas increased levels of GSH in the IFC could be a rebuttal effect after GSH consumption. These interpretations are speculative, and whether the differences that we observed are due to differential resource demands of the IFC and IPL at different points or to different default metabolic properties (perhaps derived from a different cytoarchitecture), remains to be studied.

### 4.4 Relationship between GSH modulation and cognitive performance

In young adults, an increase of GSH in IFC and IPL (from resting state MRS to fMRS) predicted a better cognitive performance. The capacity to upregulate GSH levels seems, therefore, important in the physiology sustaining cognitive control. Whereas the literature typically reports the role of GSH as an antioxidant, the results of the present work appear to tentatively show that GSH modulation is a consequence of sustained cognitive effort (previous section) and that it is associated with the quality of cognitive performance, at least in young adults (present section). We cannot make inferences about the direction or mechanisms underlying the relationship between GSH and quality of performance, but we may hypothesize that an increase in GSH could potentially relate to a reduction of oxidative stress produced by a higher neuronal/physiological metabolism that is required for successful cognitive performance. Alternatively, the balance produced by the GSH oxidative action may be crucial to preserve an (oxidant-free) proper environment for better metabolism and better cognitive performance. Furthermore, these findings reveal that baseline GSH levels and the modulatory capacity of GSH show different properties of the system, as higher baseline GSH in IPL in young adults was related to worse cognitive performance (working memory). Therefore, in contrast to baseline GSH in IPL, a higher regulatory capacity of GSH (i.e. GSH modulation as result of task performance) in young adults showed to be favorable to the organism adaptation.

In older adults, the absence of a significant statistical correlation between GSH modulation and cognitive performance could possibly be masked by certain neurodegenerative processes affecting cognitive performance.

### 4.5 Relationship between GSH modulation and blood oxygenation

Contrary to what we expected, we did not find a significant relationship between task-induced GSH modulation and the BOLD response in either region or group. It is possible that reactive stress may emerge during sustained cognitive effort, whereas the present protocol only measured the BOLD response during a short period of cognitive effort. Furthermore, GSH protects against not only reactive oxygen but also nitrogen and sulfur species (Lushchak, 2012). As such, these other GSH functions could possibly mask a relationship between oxygen consumption and GSH modulation.

### 4.6 Relationship of GSH with GABA+ and Glx

GSH showed a relationship mainly with GABA+, and the significance and direction of this relationship were dependent on age, region, and cognitive state. In young individuals, GABA+ and GSH in the IFC were not only positively correlated during the task but also co-varied (showing a parallel modulation), suggesting that they may participate in the same process. In the same sample but in the IPL region, GABA+ levels co-varied with Glx levels. As GABA and glutamate hold opposite effects modulating the neural activity (either decreasing or increasing excitability), it may be possible that the relation of these neurometabolites with GSH derives from the energy supply needed to hold either inhibitory or excitatory connections and not from the energy of neural activity itself. Both, excitatory and inhibitory connectivity require energy and consequently may be considered to produce ROS, which may be underlying the parallel regulation of GSH.

These explanations are speculative in nature and future research may dig deeper in the origin of the relationship among these neurometabolites.

A negative GSH–GABA+ relationship in IPL was observed in both groups at rest. During the task, young individuals showed the same negative correlation, but older individuals showed a positive relation with Glx instead, showing a possible age-related adaptation such that, in IPL, higher levels of GSH are associated with a lower inhibitory tone (lower GABA+ or higher Glx). However, there was no GSH–GABA+ co-variation, possibly suggesting that these metabolites hold an indirect rather than a direct relationship in the IPL.

These opposite findings regarding the GSH–GABA+ relationship in the IFC and IPL suggest that the GSH function may differ according to the region, age and cognitive state of the individual.

Finally, it is important to consider that next to the oxidative role of GSH, this metabolite has also shown to be a signaling agent leading to the release of GABA in retinal neurons to endorse cell protection (Freitas et al., 2017). In addition, GSH is synthesized from glutamate, cysteine and glicine (Bottino et al., 2021). These relationships could partially account for our observations, but given the temporal and spatial disparity between cell biological and MRS observations, it remains difficult to draw firm conclusions.

### 4.7 Limitations

One limitation of this study is the temporal disparity between the collection of fMRI and fMRS sequences. The metabolic activity during both time frames may be different due to learning, habituation and fatigue effects. This limitation could be addressed with a simultaneous MRS-fMRI acquisition (e.g., Craven et al., 2024); although to our knowledge no study has explored this approach with the measurement of GSH.

In our protocol there was a period of approximately nine minutes between the fMRI and the first resting state MRS block. Although we consider that this period could have been enough for the neurometabolic activity (occurring during the fMRI block) to return to baseline, there are no definitive parameters regarding the time that neurometabolites return to baseline levels after cognitive effort. As our study did not show neurometabolic changes in GSH (nor in GABA or Glx; Rodríguez-Nieto et al., 2024) after one block of cognitive effort, and, taking into account our counterbalanced design, it is unlikely that any possible carry-over effects impacted the present results.

It is also important to mention that *in vivo* ^1^H MRS cannot detect the oxidized form of GSH (glutathione disulfide (GSSG)) or measure the GSH/GSSG ratio (Brix et al., 2019; Satoh and Yoshioka, 2006), which is considered a more sensitive indicator of oxidative stress than GSH alone. Furthermore, the GSH levels measured by MRS do not strongly correlate with peripheral GSH levels measured through serum (Mischley et al., 2016). Therefore, our conclusions are limited only to the relationships ascribed to the reduced form of glutathione (i.e., GSH) and to the brain regions that we have specified.

Finally, although one of our exclusion criteria was the presence of psychiatric and neurological diseases, we did not screen participants for their intake of medication. We advise performing this screening in future studies.

## 5 Conclusion

This study showed a brain-region-dependent relationship of GSH with age for two critical regions of the executive control network. Resting GSH levels in the IPL were related to short-term memory processes, but the direction of this relationship was age dependent.

In regard to the modulatory properties of GSH, we observed a GSH modulation as a by-product of sustained cognitive performance in the full sample and a relationship between GSH modulation and quality of cognitive performance in young adults. Once more, these findings were brain region dependent. Altogether, this suggests that the functionality of GSH is dependent on age and brain region, reflecting different physiological properties.

Regarding the relationship of GSH with physiological properties, we did not observe a relationship with the BOLD response. Finally, we observed a relationship between GABA and GSH, which was age and brain-region-dependent.

These findings deepen our understanding of the physiology of GSH, its relation with age, cognition and other metabolites.

## Data Availability

The datasets presented in this study can be found in online repositories. The names of the repository/repositories and accession number(s) can be found at: https://osf.io/7ngrw/overview?view_only=07370c3614a545389b0b8915f5f10243.
