# Supplementary material for: The role of glutathione in cognition, cognitive effort, and cognitive endurance in young and older adults
Source: Front Aging Neurosci. 2026 Feb 27;18:1729015. doi: 10.3389/fnagi.2026.1729015 (PMC12982376; doi:10.3389/fnagi.2026.1729015)
Supplement: Supplementary file 1 [file Data_Sheet_1.docx]

Supplementary Material

**Table S1.** Descriptive statistics of baseline GSH levels according to order of presentation.

| GSH IFC baseline | IFC first block | IFC second block |
| --- | --- | --- |
| Young | 1.35(.24)  .95-1.78 | 1.23 (.31)  .57-1.69 |
| Old | 1.79 (.87)  .75-4.22 | 1.65 (.95)  .54-4.74 |
| GSH IPL baseline | IFC first block | IFC second block |
| Young | 1.75 (.96)  .82 – 4.61 | 2.21 (.1.11)  .69 – 4.63 |
| Old | 2.16 (.89)  1.07-4.16 | 2.19 (.89)  .48-3.93 |

**Effects of time (order) on GABA and Glx**

**Figure S1**. Null effects of time (scanning order) on GSH baseline levels.


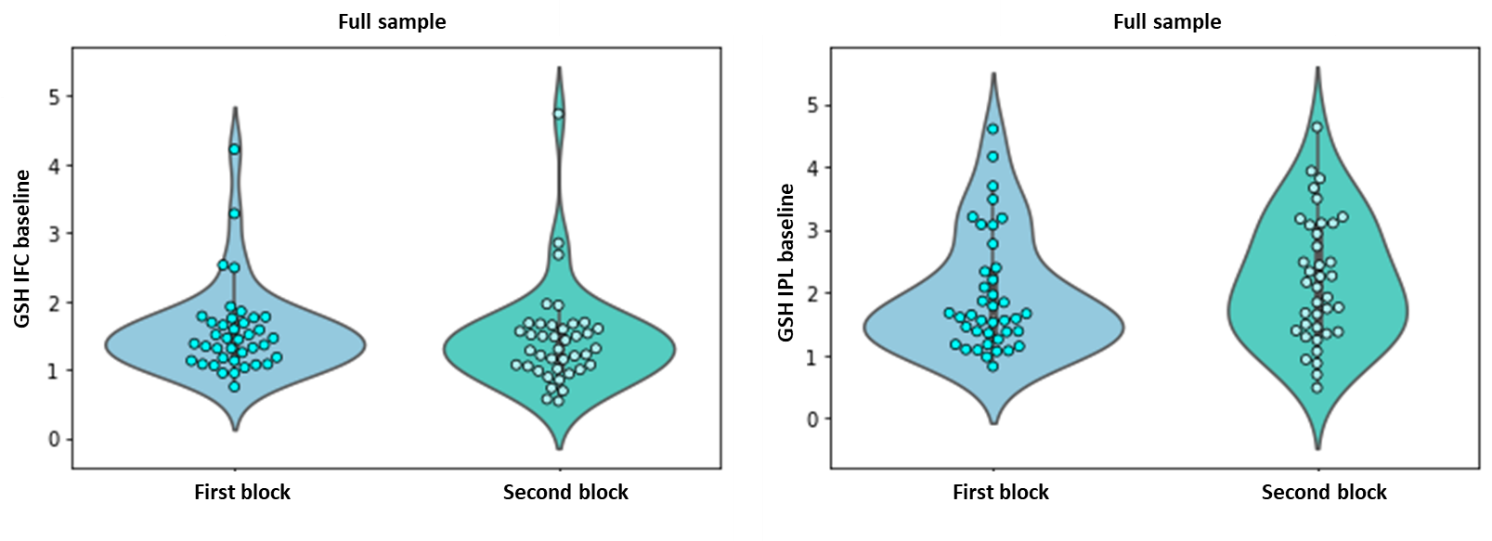


**Table S2.** GABA levels according to the scanning order in the baseline condition.

| GABA IFC baseline | IFC first block | IFC second block |
| --- | --- | --- |
| Young | 2.49 (.34)  1.84 – 3.41 | 2.55 (.45)  1.26-3.61 |
| Old | 2.19 (.33)  1.67 – 3.03 | 2.31 (.53)  1.65 – 3.63 |
| GABA IPL baseline | IPL first block | IPL second block |
| Young | 2.35 (.27)  1.85 – 2.69 | 2.18 (.23)  1.83-2.76 |
| Old | 2.12 (.29)  1.56 – 2.64 | 1.92 (.25)  1.32 – 2.34 |

*GABA Baseline.* The GLM did not show order effects nor an interaction with Age on baseline GABA levels in the IFC (R^2^=.11; Order: F_(1,80)_=.91, p=.34; Order X Age: F_(1,80)_=.07, p=.79; Figure S1; Table S2). However, in the baseline GABA levels in IPS an order effect was observed with no interaction with age (R^2^=.26; Order: F_(1,79)_=9.66, p=.003; Order X Age: F_(1,79)_=.06, p=.81). These results showed that GABA baseline levels were higher during the first block of baseline in the full sample (Figure S1; Table S2).

**Table S3.** GABA levels according to the scanning order in the task condition.

| GABA IFC task | IFC first block | IFC second block |
| --- | --- | --- |
| Young | 2.41 (.45)  1.51 – 3.08 | 2.38 (.39)  1.78-3.43 |
| Old | 2.26 (.46)  1.48 – 3.31 | 2.21 – .39  1.34-2.77 |
| GABA IPL task | IPL first block | IPL second block |
| Young | 2.11 (.28)  1.65 – 2.79 | 2.32 (.22)  1.94-2.86 |
| Old | 2.01(.25)  1.58 –2.41 | 2.02 (.25)  1.53 – 2.65 |

*GABA task.* The GLM did not show order effects nor an interaction with Age on GABA levels in the IFC during the task (R^2^=.04; Order: F_(1,79)_=.02, p=.89; Order X Age: F_(1,79)_=.19, p=.66; Figure S3). In contrast, in the IPS an Order effect was observed indicating that GABA levels were higher when being measured during the second block of the task. No interaction with Age was observed (R^2^=.21; Order: F_(1,80)_=4.29, p=.04; Order X Age: F_(1,80)_=3.06, p=.08; Figure S3; Table S3).

**Figure S2.** Effects of scanning order on GABA levels during baseline and task performance.


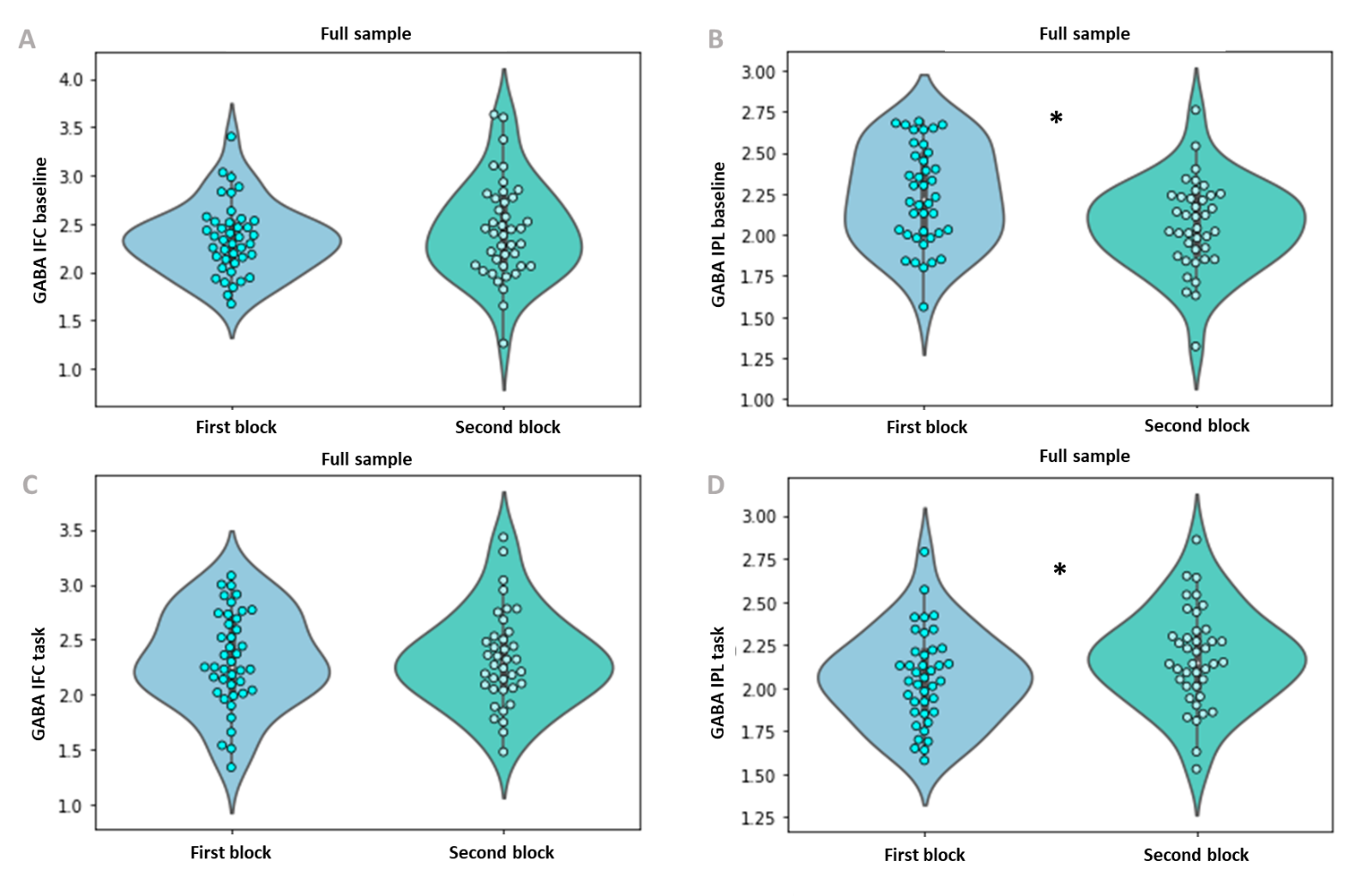


**Table S4**. Glx levels according to the screening order in the baseline condition.

| Glx IFC baseline | IFC first block | IFC second block |
| --- | --- | --- |
| Young | 7.11 (.76)  5.71 – 8.57 | 7.17 (.79)  5.26-8.98 |
| Old | 6.55 (.74)  5.03 – 8.15 | 6.67 (.72)  5.42 – 8.31 |
| Glx IPL baseline | IPL first block | IPL second block |
| Young | 7.06 (.54)  6.10 – 8.15 | 6.47 (.72)  5.57-7.86 |
| Old | 6.21 (.75)  4.72 –7.88 | 6.31 (.71)  5.27 – 7.99 |

*Glx Baseline.* The GLM did not show order effects nor an interaction with Age on baseline Glx levels in the IFC (R^2^=.12; Order: F_(1,80)_=.34, p=.6+; Order X Age: F_(1,80)_=.02, p=.88; Figure S2). However, in the baseline Glx levels in IPL an order effect on its interaction with age was observed (R^2^=.19; Order: F_(1,79)_=2.66, p=.11; Order X Age: F_(1,79)_=4.74, p=.03). Posthoc analyses indicated that Glx levels were higher in the st block of baseline in young adults (Figure S2).

**Table S5**. Glx levels according to the scanning order in the task condition.

| Glx IFC task | IFC first block | IFC second block |
| --- | --- | --- |
| Young | 6.89 (.61)  5.91 – 7.95 | 7.37 (.88)  5.72-8.75 |
| Old | 6.62 (.83)  5.16 – 7.75 | 6.54 (.81)  5.54 – 9.92 |
| Glx IPL task | IPL first block | IPL second block |
| Young | 6.67 (1.01)  4.95 – 8.46 | 6.74 (.62)  4.88 – 7.77 |
| Old | 6.36 (.99)  4.76 – 8.95 | 6.06 (.71)  4.36 – 7.14 |

*Glx Task.* No effects of Order, nor of its interaction with Age were found for Glx levels during the task in either region (IFC: R^2^=.15; Order: F_(1,80)_=1.27, p=.26; Order X Age: F_(1,80)_=2.51, p=.12; IPL: R^2^=.09; Order: F_(1,80)_=.39, p=.53; Order X Age: F_(1,79)_=.98, p=.32).

**Figure S3.** Effects of scanning order on Glx levels during baseline and task performance


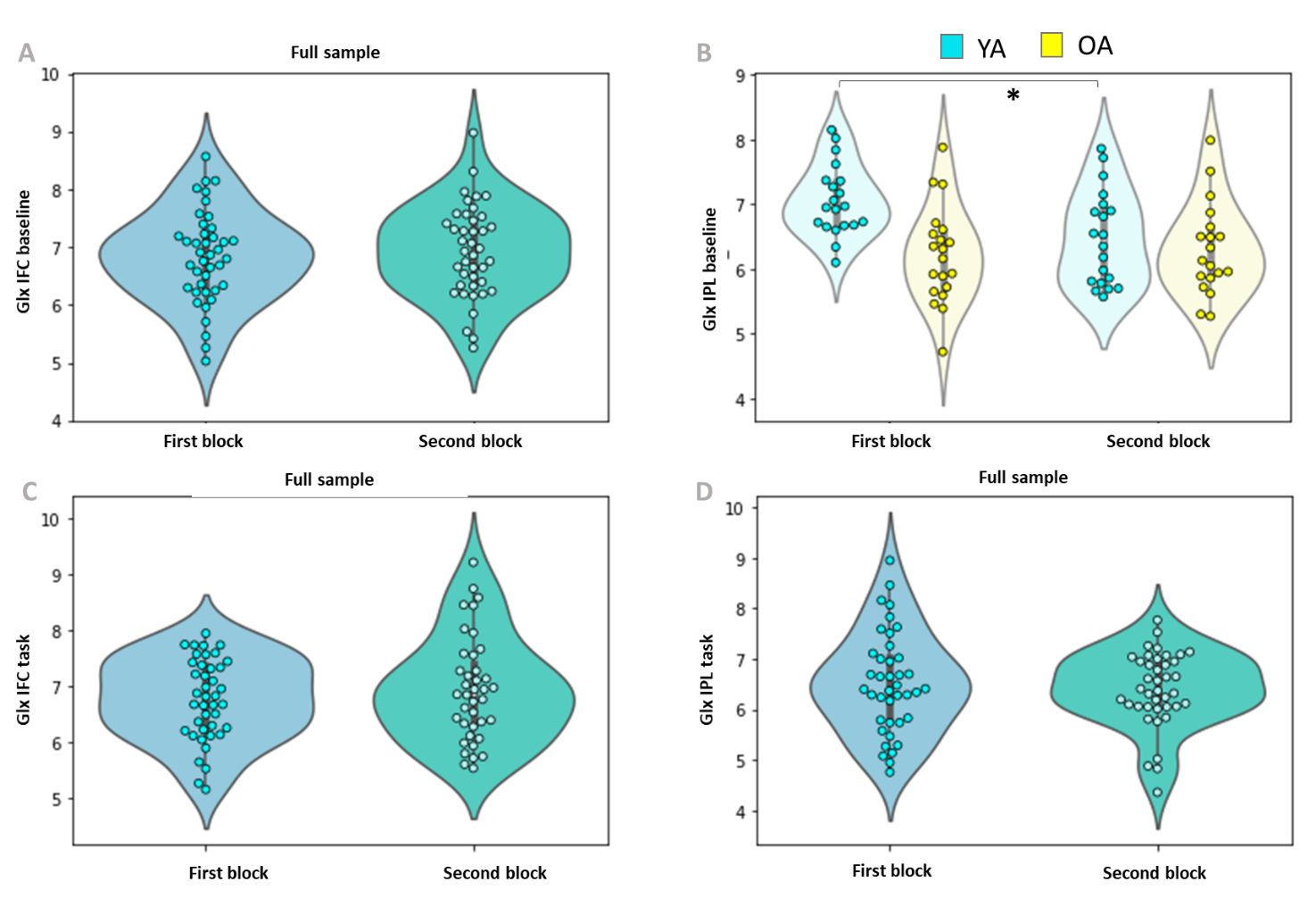


**NEUROMETABOLIC NETWORKS – Correlation matrices**

**Table S7.** Partial correlations controlling for gray and white matter from that specific region (IFC in young adults)

| **IFC baseline**  **YOUNG** | | | | | | |
| --- | --- | --- | --- | --- | --- | --- |
|  | GABA | Glx | GSH | NAA | Cho | Cr |
| GABA | 1 | .26  (.11) | .31  (.06) | .06  (.71) | -.02  (.92) | -.05  (.76) |
| Glx |  | 1 | .04  (.77) | .36*  (.02) | .37*  (.02) | .32  (.04) |
| GSH |  |  | 1 | -.26  (.11) | -.38  (.01)* | -.16  (.32) |
| NAA |  |  |  | 1 | .49  (.001)* | .62  (<.001)* |
| Cho |  |  |  |  | 1 | .54  (.001)* |
| Cr |  |  |  |  |  | 1 |
| **IFC task**  **YOUNG** | | | | | | |
|  | GABA | Glx | GSH | NAA | Cho | Cr |
| GABA | 1 | .05  (.76) | .42 *  .007 | .07  (.68) | -.05  (.74) | .19  (.24) |
| Glx |  | 1 | .26  (.11) | .19  (.23) | .46*  (.003) | .32  (.04) |
| GSH |  |  | 1 | -.19  (.23) | -.11  (.49) | .11  (.49) |
| NAA |  |  |  | 1 | .29  (.07) | .37  (.02) |
| Cho |  |  |  |  | 1 | .63*  (<.001) |
| Cr |  |  |  |  |  | 1 |
| **IFC diff**  **YOUNG** | | | | | | |
|  | GABA | Glx | GSH | NAA | Cho | Cr |
| GABA | 1 | -.15  (.36) | .39*  (.01) | -.17  (.3) | -.22  (.17) | -.31  (.05) |
| Glx |  | 1 | .02  (.91) | .12  (.46) | .07  (.65) | .27  (.1) |
| GSH |  |  | 1 | -.28  (.08) | -.12  (.45) | .12  (.47) |
| NAA |  |  |  | 1 | .51*  (<.001) | .42*  (.008) |
| Cho |  |  |  |  | 1 | .72*  (<.001) |
| Cr |  |  |  |  |  | 1 |

(*) Comparisons surviving FDR correction

**Table S8.** Partial correlations controlling for gray and white matter from that specific region (IPL in young adults)

| **IPL baseline**  **YOUNG** | | | | | | |
| --- | --- | --- | --- | --- | --- | --- |
|  | GABA | Glx | GSH | NAA | Cho | Cr |
| GABA | 1 | .03  (.81) | -.47*  (.003) | .17  (.31) | -.05  (.74) | -.02  (.91) |
| Glx |  | 1 | .04  (.81) | -.01  (.92) | .03  (.84) | -.17  (.29) |
| GSH |  |  | 1 | -.11  (.51) | .26  (.12) | .37  (.02) |
| NAA |  |  |  | 1 | .06  (.37) | .29  (.07) |
| Cho |  |  |  |  | 1 | .45*  (.004) |
| Cr |  |  |  |  |  | 1 |
| **IPL task**  **YOUNG** | | | | | | |
|  | GABA | Glx | GSH | NAA | Cho | Cr |
| GABA | 1 | .21  (.22) | -.43*  (.006) | .04  (.81) | .06  (.71) | -.24  (.14) |
| Glx |  | 1 | .01  (.98) | -.11  (.55) | -.01  (.95) | -.01  (.97) |
| GSH |  |  | 1 | -.17  (.29) | .25  (.12) | .42*  (.009) |
| NAA |  |  |  | 1 | -.24  (.15) | .01  (.96) |
| Cho |  |  |  |  | 1 | .38  (.02) |
| Cr |  |  |  |  |  | 1 |
| **IPS diff**  **YOUNG** | | | | | | |
|  | GABA | Glx | GSH | NAA | Cho | Cr |
| GABA | 1 | -.39*  (.01) | -.09  (.58) | .17  (.31) | .12  (.45) | .18  (.28) |
| Glx |  | 1 | .38*  (.02) | -.15  (.36) | -.29  (.08) | -.13  (.42) |
| GSH |  |  | 1 | -.13  (.42) | .06  (.72) | .38*  (.02) |
| NAA |  |  |  | 1 | .64*  (<.001) | .34  (.03) |
| Cho |  |  |  |  | 1 | .59*  (<.001) |
| Cr |  |  |  |  |  | 1 |

(*) Comparisons surviving FDR correction

**Table S9.** Partial correlations controlling for gray and white matter from that specific region (IFC in older adults)

| **IFC baseline**  **OLD** | | | | | | |
| --- | --- | --- | --- | --- | --- | --- |
|  | GABA | Glx | GSH | NAA | Cho | Cr |
| GABA | 1 | -.17  (.28) | .29  (.09) | -.02  (.91) | .11  (.54) | -.07  .66 |
| Glx |  | 1 | .17  (.33) | .03  (.84) | .12  (.45) | .13  (.43) |
| GSH |  |  | 1 | -.03  (.83) | .38  (.03) | .25  (.15) |
| NAA |  |  |  | 1 | -.09  (.56) | .41  (.01) |
| Cho |  |  |  |  | 1 | .24  (.14) |
| Cr |  |  |  |  |  | 1 |
| **IFC task**  **OLD** | | | | | | |
|  | GABA | Glx | GSH | NAA | Cho | Cr |
| GABA | 1 | -.26  (.13) | -.16  (.35) | .18  (.28) | .19  (.27) | .29  (.08) |
| Glx |  | 1 | .29  (.09) | .16  (.32) | .100  (.56) | .33  (.05) |
| GSH |  |  | 1 | -.01  (.92) | .01  (.9) | .22  (.21) |
| NAA |  |  |  | 1 | .07  (.67) | .47*  (.003) |
| Cho |  |  |  |  | 1 | .31  (.06) |
| Cr |  |  |  |  |  | 1 |
| **IFC diff**  **OLD** | | | | | | |
|  | GABA | Glx | GSH | NAA | Cho | Cr |
| GABA | 1 | .06  (.74) | .06  (.74) | .04  (.84) | .15  (.42) | .13  (.51) |
| Glx |  | 1 | .21  (.28) | .13  (.49) | -.02  (.92) | -.11  .61 |
| GSH |  |  | 1 | -.08  (.69) | -.25  (.18) | .19  (.31) |
| NAA |  |  |  | 1 | .68*  <.001 | .62*  <.001 |
| Cho |  |  |  |  | 1 | .64*  <.001 |
| Cr |  |  |  |  |  | 1 |

(*) Comparisons surviving FDR correction

**Table S10.** Partial correlations controlling for gray and white matter from that specific region (IPL in older adults)

| **IPL baseline**  **OLD** | | | | | | |
| --- | --- | --- | --- | --- | --- | --- |
|  | GABA | Glx | GSH | NAA | Cho | Cr |
| GABA | 1 | -.31  (.06) | -.48*  (.003) | .44*  (.007) | .23  (.18) | .29  (.08) |
| Glx |  | 1 | .15  (.38) | -.22  (.19) | -.02  (.87) | .05  (.78) |
| GSH |  |  | 1 | -.22  (.19) | -.11  (.49) | -.06  (.71) |
| NAA |  |  |  | 1 | .42*  (.01) | .67*  (<.001) |
| Cho |  |  |  |  | 1 | .46*  (.005) |
| Cr |  |  |  |  |  | 1 |
| **IPL task**  **OLD** | | | | | | |
|  | GABA | Glx | GSH | NAA | Cho | Cr |
| GABA | 1 | -.05  (.75) | -.19  (.24) | .18  (.27) | .08  (.63) | .25  (.13) |
| Glx |  | 1 | .51*  (.001) | -.04  (.79) | -.06  (.72) | .18  (.25) |
| GSH |  |  | 1 | -.25  (.12) | .11  (.49) | .11  (.53) |
| NAA |  |  |  | 1 | .34  (.03) | .54*  (<.001) |
| Cho |  |  |  |  | 1 | .48*  (.002) |
| Cr |  |  |  |  |  | 1 |
| **IPL diff**  **OLD** | | | | | | |
|  | GABA | Glx | GSH | NAA | Cho | Cr |
| GABA | 1 | -.04  (.81) | .04  (.81) | -.02  (.88) | -.11  (.51) | -.06  (.71) |
| Glx |  | 1 | -.22  (.21) | .34  (.04) | .29  (.08) | .17  (.31) |
| GSH |  |  | 1 | -.45*  (.006) | -.49*  (.002) | -.28  (.08) |
| NAA |  |  |  | 1 | .81*  (<.001) | .66*  (<.001) |
| Cho |  |  |  |  | 1 | .71*  (<.001) |
| Cr |  |  |  |  |  | 1 |

(*) Comparisons surviving FDR correction

**Table S11.** Comparison between correlations from young and older people (from where at least one of the two coefficients was significant).

|  |  | **Young** | | **Old** | |  |  |
| --- | --- | --- | --- | --- | --- | --- | --- |
| **Metabolite** | **Condition** | **Pearson**  **r** | p | **Pearson**  **r** | p | t | **P value** |
|  | **Age differences within intrarregional networks** | | | | | | |
| **Region** | **Metabolites** | **Pearson**  **r** | p | **Pearson**  **r** | **N** | t | **P value** |
| IFC-baseline | GSH-Cho | -.38 | .01 | .38 | .03. | -3.35 | .0007 |
| IFC-task | GABA-GSH | .42 | .007 | -.16 | 35 | 2.54 | .01 |
| IPL-baseline | NAA-Cr | .29 | .07 | .67 | .001 | -2.19 | .03 |
| IPL-task | Glx-GSH | .01 | .98 | .51 | .001 | -2.36 | .02 |
| IPL-task | NAA-Cr | .01 | .96 | .54 | <.001 | -2.55 | .01 |
| IPL-diff | Glx-GSH | .38 | .02 | -.22 | .21 | 2.64 | .008 |
| IPL-diff | GSH-Cho | .06 | .72 | .49 | .002 | 2.02 | .04 |
| IPL-diff | GSH-Cr | .38 | .02 | -.28 | .08 | 2.91 | .003 |
